# A Complementary Intervention to Promote Wellbeing and Stress Management for Early Career Teachers

**DOI:** 10.3390/ijerph18126320

**Published:** 2021-06-11

**Authors:** Stevie-Jae Hepburn, Annemaree Carroll, Louise McCuaig-Holcroft

**Affiliations:** 1 School of Education, The University of Queensland, Brisbane 4067, Australia; a.carroll@uq.edu.au; 2 School of Human Movement and Nutrition Sciences, The University of Queensland, Brisbane 4067, Australia; l.mccuaig@uq.edu.au

**Keywords:** wellbeing, teacher stress, yoga, perceived stress, stress management, health, positive psychology, mindfulness

## Abstract

The educational climate and culture in our schools present a variety of environmental (contextual) factors that influence teacher wellbeing, job satisfaction, and work-related stress. The magnitude of contextual factors cannot be ignored, and directing attention towards the environment teachers face daily is essential. Primary (organisational)-level interventions are documented in organisational health and wellbeing literature; however, to provide teachers with stress management strategies for promoting wellbeing, attention must also be directed towards secondary (individual)-level interventions. The present study addressed the issue of stress management techniques for early career teachers (*n* = 24) and aimed to contribute to the research surrounding complementary interventions (CIs) for educators. The intervention was designed to include strategies that operated through cognitive and physiological mechanisms that regulated the stress response and increased awareness of behaviours, emotions, and reactivity. The self-report measures included perceived stress, attention awareness, subjective wellbeing, burnout, and job-related affective wellbeing. The results indicated a statistically significant decrease in perceived stress and increases in attention awareness and subjective wellbeing. The salivary cortisol levels (waking and resting) decreased from baseline to week 6, and the pre- and post-session salivary cortisol levels indicated an immediate decrease in cortisol for weeks 4 to 6.

## 1. Introduction

Teacher education programs focus almost exclusively on pedagogical, procedural, and conditional knowledge with the exclusion of the highly emotional experiences of teaching and guidance surrounding the importance of establishing and negotiating relationships. As a result, early career teachers (ECTs) are left feeling that they are unprepared for the role, and this can exacerbate their fears and concerns [1]. ECTs can experience feelings of uncertainty, confusion, and self-doubt [2,3]; hence, it is not surprising that ECTs are more susceptible to work stress. ECTs can experience insecurities regarding the multiple roles required as a teacher while simultaneously learning how to be a teacher. Additionally, there are no fixed schedules, and as a result, ECTs may not know when they have done enough. Consequently, individuals who are high achievers or strive to create excellent lesson activities can become vulnerable to over-exertion [1]. Teacher stress and wellbeing is a vast issue that has implications for not only individual teachers and their families but also the students in their care, the school culture, and the education profession.

A key aspect of the present study is the notion of promoting integrated (holistic) wellbeing and providing strategies and tools for stress management. The system of yoga is presented as a set of tools or techniques that encourage the development of resources to assist individuals with stress management, and the intervention has been designed to promote wellbeing and health through multiple techniques that relate to psychological (mental), physiological, and social (interpersonal) wellbeing.

### 1.1. Background

#### Organisational Wellbeing Initiatives

Due to the extensive impact of workplace stress, occupational stress and preventative stress management interventions have received attention from not only academic scholars, but also government and industry bodies. As outlined by the World Health Organization [4], the workplace provides the setting for the promotion of health activities, including physical and psychological wellbeing. Stress management interventions are classified as either primary, secondary, or tertiary [5]. Primary interventions aim to address the sources of stress within the workplace at the organisation or group level, focus on organisational restructuring or development, and occur over a longer period (e.g., a 12-month period). Secondary interventions are aimed at the individual level and include somatic, cognitive, or multimodal techniques, for example, breathing techniques and reducing physical tension (progressive muscle relaxation). Tertiary interventions aim to ameliorate existing stress and symptoms for individuals within the organisation, for example, counselling and employee assistance programs [5,6]. Cognitive interventions refer to mindfulness-based techniques and reducing negative thoughts, whereas multimodal interventions incorporate both somatic and cognitive techniques [7]. It was beyond the scope of the present study to investigate a primary (organisation)-level intervention, nor was the aim to promote a single solution for the multifaceted issue of teacher stress. Rather, the present study explored the implementation of a secondary-level complementary intervention (CI) for educators.

In Australia, there are ongoing developments in the area of ECT support and teacher wellbeing initiatives across different jurisdictions. At present, the Australian Professional Standards for Teachers [8] from the Australian Institute for Teaching and School Leadership do not explicitly refer to wellbeing support for teachers. Implementing wellbeing initiatives is determined by the individual school leaders. Since this study was completed, there have been developments within the field of teacher wellbeing, and post-COVID-19, it could be expected that further developments will continue to remain a priority for education systems. For example, the Staff Wellbeing Framework from the Department of Education, Queensland, provides a model for wellbeing including five domains: physical, financial, occupational, psychological, and social and community engagement. However, to date, no primary (department or jurisdiction)-level interventions have been reported.

Interventions that focus solely on the primary (organisation) level may not be as effective for enhancing wellbeing as individual-level (secondary) approaches [9]. When evaluating stress management interventions, Cooper and colleagues [10] explain that the effectiveness of stress management training is limited when the stressors are systemic or structural (e.g., excessive workload); therefore, the coping abilities of individuals may be insufficient long-term when role re-structuring or job redesign are required. Role re-structuring and job re-design are classified as primary-level stress management interventions. An additional argument for the use of secondary interventions as opposed to primary interventions is that it is unrealistic to expect a primary intervention to remove all the potential stressors from an organisation [10]. It is important to note, however, that job redesign may not be equally achievable across all professional contexts. For example, there are limitations surrounding adjusting contact hours for teachers, whereas in other professions (e.g., nursing or occupational therapy), such changes are feasible. Consequently, it does not appear realistic to simply suggest role restructuring alone as the ‘solution’ for teacher stress. Therefore, it is assumed that multimodal secondary interventions, as included in the present study, can capitalise on the strengths of both the somatic and cognitive approaches and render the greatest stress management potential [7].

In recent years, the development of mindfulness-based interventions (MBIs) for educators has been an area of rapid growth; however, there have been limited yoga-based CIs for educators. The key enhancement terms in research investigating MBIs for teachers are *teacher wellbeing* determined through quantitative measures assessing perceived stress, burnout, mindfulness, and loving-kindness; *experiences and practising mindfulness* explored through qualitative techniques (semi-structured interviews); and *teacher performance* determined by self-efficacy, classroom teaching, and student behavioural outcomes [11]. Based on the existing literature, mindfulness meditation is confirmed as effective in reducing threats to teacher wellbeing, and positive effects include improved wellbeing, reduced perceived stress, improved physiological symptoms of stress (cortisol levels and blood pressure), improved sleep quality, and reduction in psychological distress [11]. Mindfulness training improves teacher–student communication and interactions [12] and promotes prosocial interaction [13]. For example, the mindfulness facets of *non-judgement* and *observing* are proposed to improve a teacher’s ability to respond to their students’ needs [14]. The current range of MBIs and CIs for educators provide valuable support for teachers and for promoting teacher wellbeing through the use of mindfulness-based strategies.

The Mindfulness-Based Stress Reduction (MBSR) program developed by Kabat-Zinn [15] has been trialed with school teachers (see for example, [16,17]), indicating improvements in perceived stress, wellbeing, mindfulness, depression, and anxiety. Similarly, studies utilising a modified MBSR (mMBSR) program [18,19] have reported significant reductions in psychological symptoms of burnout and increased self-compassion and trait mindfulness. Subsequently, there has been an increase in MBIs that closely align with the original MBSR implemented as 8-week [13,20], 9-week [21], and 10-week programs [22]. Alternative structures for MBIs that include some components of the MBSR have been conducted over longer periods, for example, 12-month programs with bimonthly sessions [23], 3 years of training (pre-service period) coupled with an 8-week intensive program (first year of teaching) [24] and four full-day sessions completed over a 6-week period [25]. Even though the method of instruction and delivery of the interventions have varied, the mindfulness-based strategies are consistent, and the participant group discussion and related activities have been adjusted for school-based settings.

Pilot studies for MBIs specifically designed for teachers, for example, the Cultivating Awareness and Resilience in Education (CARE) program [26], have paved the way for larger studies indicating consistently significant results for stress reduction and cultivating mindfulness in teachers and students (see [26,27]). The CARE program (30 h), delivered as five full-day (6-h) sessions five to six weeks apart, is specifically designed for application in the classroom. For example, the activities are cognitively based: ‘caring practice’ is a short period of reflection to focus on feelings of kindness for oneself and others, and ‘mindful listening’ involves listening to another and noticing emotional reactions. The practices assist the teachers with conflict resolution and behaviour management concerns. The professional development modules combine direct instruction of the mindfulness-based skills, practice, reflection activities, group discussion, and home practice [28]. Based on the information presented by the authors, the program does not include any relaxation activities, postures, or poses to reduce the stress response in the body or direct instruction (theory) regarding the impact of stress.

Similarly, the Stress Management and Relaxation Techniques in education (SMART) program [29] supports the benefits of MBIs for educators. The SMART program is an 8-week professional development program (36 contact hours), including mindfulness training. The five main teaching activities are designed to develop mindfulness skills and promote self-compassion in teachers’ professional lives. Additionally, lectures are included to highlight how mindfulness can be used to regulate emotions and stress. The randomised control trials for the SMART program did not report any physiological changes (blood pressure or cortisol); however, there were positive psychological effects (lowered perceived stress, anxiety, depression, and burnout and increased mindfulness, attention, and self-compassion) [30].

The Community Approach to Learning Mindfully (CALM) program for educators [30] aimed to create a culture of wellbeing and self-care within the school community. The 64 intervention sessions were 20 min in length held before the start of the school day four days a week for 16 weeks. The manualised program featured a thematic focus for each week, and the postures and practices reflected the theme of the week. The individual session structure mirrored that of an integrated yoga class. Interestingly, there were both physiological and psychological changes experienced by the participants. Reported changes in psychological measures included increased mindfulness, positive affect, and classroom management. Physiological measures included decreased blood pressure and cortisol awakening response. The CALM program was designed to increase accessibility and promote skill transfer; that is, the skills and practices were designed to be extended to other contexts and situations.

Previous quantitative studies (e.g., [13,17,18,30]) have assessed the impact of CIs on educators incorporating diverse outcome measurements—for instance, self-report measures to assess mindfulness, self-efficacy, perceived stress, burnout, time urgency, and classroom management and biological measures (e.g., cortisol awakening response) to assess physiological responses to stress and sleep-related impairment. However, participant perspectives are not included (e.g., interviews or reflections), and qualitative studies [31] have relied on interviews alone to determine the impact of integrating CIs into teacher training education. The present study included a mixed-methods design; however, due to the large volume of data collected, the qualitative datasets will not be reported in this paper. The approach in the present thesis was not to examine the use of mindfulness-based strategies or the application of the Mindfulness-Based Stress Reduction (MBSR) program [15]. Rather, the overarching approach was two-fold; first, decrease the stress response (physical component), and second, provide self-knowledge and understanding of the importance of prioritising wellbeing (theoretical component). As a result, the study makes a valuable contribution to the field of research surrounding CIs for educators.

### 1.2. Yoga and Stress Management

There is an extensive list of the benefits of yoga presented in the literature and a growing body of research in neuroscience surrounding the mechanisms and benefits of yoga practice. A yoga practice that includes postures and breathing practices is classified as *Hatha* yoga. ‘*Ha*’ symbolises the Moon and the parasympathetic nervous system, whereas ‘*tha*’ symbolises the Sun and the sympathetic nervous system. The focus in *Hatha* yoga is balancing bodily responses [32]. Yogic philosophy indicates that there are three main layers of tension, namely, muscular (physical), emotional, and mental. Table 1 has been created to summarise the techniques practiced in yoga and the stress response. The techniques listed in Table 1 are from the “eight-limbed” system of yoga called the Ashtanga Yoga System (*ashta*: eight, *anga*: limb). In the *Yoga Sutras* of Patanjali, the 195 sutras were grouped to form the Ashtanga Yoga System (see [33]). Unfortunately, a detailed account of the rich, fascinating history of yoga is beyond the scope of the present study. For additional information surrounding the history and evolution of yoga up to the modern era, see Sovik and Bhavanani [34] and Pradhan [32].

The techniques are practised to dissolve or reduce the layers of tension. Muscular tension refers to the nervous system and endocrinal imbalances. Emotional tension results from an inability to express or acknowledge emotions (repression). Mental tension is accumulated through experience [35]. In Table 1, each of the layers of tension is aligned with the techniques, underlying mechanisms, and outputs, as detailed in the literature. It is difficult to isolate a specific mechanism, because the techniques are typically practised in unison. Therefore, it is difficult to represent the application of the system of yoga visually, because there is a large amount of overlap and interaction between the different layers, techniques, and mechanisms. It is not a straight linear or cause-and-effect process. Due to the interconnected nature of the layers of tension and the techniques, there is a degree of bidirectional (top-down and bottom-up) feedback and feedforward that occurs. In integrated yoga practice (included in the study intervention), all of the techniques are included, therefore creating a holistic approach to dissolving tension at all three levels. The specific outputs in relation to the stress response are detailed in the following section.

#### Yoga and the Stress Response

Increased hypothalamus–pituitary–adrenal axis (HPA-axis) activity is assessed by cortisol levels, and it is commonly used as a measure of biological stress. Kamei et al. [36] indicated that cortisol levels decreased and alpha wave levels increased after only 120 min of Hatha yoga practice (*Asana*, *Pranayama*, and focused attention meditation) in experienced yoga practitioners. Early research has utilised biological measures such as salivary cortisol to measure the impact of yoga practice as an adjunct treatment for depression (see [37]). Some argue there are inconsistencies in the research findings [38]; however, there are several recent studies presenting significant findings (e.g., [39,40,41]).

The activation of the vagal nerve is a proposed mechanism of yoga practice. The afferent fibres of the abdominal branches of the vagal nerve communicate essential peripheral information via central reflex pathways, which initiate endocrine, autonomic, and behavioural responses [42,43]. Diaphragmatic breathing influences vagal tone and thus stimulates the parasympathetic system, which can result in a reduction in the stress response and neuroendocrine release of hormones (e.g., cortisol via the HPA-axis) [44]. Many yoga postures promote abdominal tone and internal muscle activation, and consequently, afferent vagal stimulation [45]. For example, Iyengar Yoga poses thought to alleviate feelings of depression have been investigated, with findings indicating a significant decrease in self-reported depression, trait anxiety, fatigue, and negative mood [46]. Specific poses have been associated with increased cardiac vagal modulation and heart rate variability (changes in the heart’s beat-to-beat intervals) [47], and vagal tone influences heart rate variability (HRV).

When vagal tone is increased, heart rate and blood pressure decrease. Vagal control of heart rate and HRV are more rapid responses than the sympathetic nervous system (SNS), therefore allowing more flexibility in response to stressors or challenges. If an individual is maintaining stability through allostasis, there is a decrease in the flexibility of the stress-response system and increased risk to the individual (allostatic load [48]). Yoga practices can promote vagal tone and stimulate the parasympathetic nervous system (PNS) (illustrated as an ‘output’ in Table 1) via diaphragmatic breathing [49], voluntary controlled breathing patterns [48], and physical postures that are practised with non-reactive awareness [45] (illustrated as techniques and mechanisms in Table 1). The practices also increase vagal nerve stimulation and, consequently, GABA levels in the brain. Therefore, the practices that support vagal tone can downregulate allostatic load, the SNS, and the HPA-axis [50], which can have negative psychological and physiological impacts.

Yoga-based practices influence both top-down (cognitive) and bottom-up (physiological) processes. Bottom-up processes include vagal afference, which significantly contributes to neuroception. Neuroception refers to the physiological states that can be elicited by environmental factors [43]. The stimulation of the PNS via vagal pathways increases GABA transmission, HPA-axis regulation, and the anterior cingulate (executive function); consequently, there is a decrease in the reactivity or overactivity of the amygdala. It is also suggested that prosocial hormones (prolactin, oxytocin) are increased by vagal activation, therefore contributing to positive emotions (empathy, bonding, and affection), often reported by yoga practitioners [51].

In an integrated yoga practice, the ethical aspects are synergised with the meditation practice and promote pro-social behaviour. It is the concentrative meditation (focused attention) practices that encourage the practitioner to see the conditions for creating or relieving mental and emotional suffering, which is referred to as experiencing fluctuations of the mind [52] (p. 5). Yoga practices facilitate bidirectional feedback through the process of integrating top-down (high-level brain networks) and low-level brain networks and bottom-up mechanisms. Within the context of stress management maintaining attentional stability (cultivated through yoga practice, *Dharana,* and *Samyama* in Table 1) can reduce negative reappraisal and rumination due to the engagement with and awareness of sensations from the body [53]. Attentional stability contributes to cognitive factors (e.g., response inhibition and selective attention) and results in a reduction in habitual maladaptive tendencies (autonomic and behavioural).

Through the yoga practices outlined above, lower-level brain networks are activated (i.e., PSN and vagal tone), therefore counteracting the activation of the SNS such as inflammation, muscle tension, and pulmonary constriction (breathing changes) [52]. Breathing practices (*Pranayama*) in particular can rapidly normalise the SNS [54]. Interoceptive messages travelling from the body to the regulatory brain centres via the thoracic vagal nerves are altered via changes in breathing patterns. The sensors within the respiratory system (e.g., nasal passages, throat, lungs, and diaphragm) communicate with the regulatory brain centres, resulting in rapid changes to attention, behaviour, emotion regulation, and perception [51]. The integration of brain regions results from the strengthening of executive monitoring systems, which is a proposed mechanism of mindfulness-based meditative practices [55]. Bottom-up mechanisms are integrated through periphery sensory feedback (muscular–skeletal and heart rate variability), resulting in less disruption to bodily systems from the acute and chronic stress response, for example, muscular–skeletal tensions and neuroendocrine disruption.

In summary, it is proposed that through integrative yoga practice, interoceptive experience provides integration across high-level and low-level brain regions, consequently assimilating homeostatic, visceral, sensory, environmental, and social feedback and feedforward, which improves accuracy in detecting and responding to perceived threats and reducing the impact of chronic stress [52]. Put simply, the techniques have underlying mechanisms that provide the opportunity to reduce the stress response and dissolve the physical, mental, and emotional layers of tension that accumulate.

Often the terms yoga, meditation, and mindfulness are used interchangeably. The system of yoga includes mindfulness practices (e.g., focused attention (FA) and open monitoring (OM) meditation, presented centred awareness), which are included in the present study through the techniques and mechanisms outlined in Table 1. In fact, research surrounding body awareness and mindfulness has indicated that the same mechanisms may operate in yoga and mindfulness-based interventions [52].

Early career teachers can be vulnerable to feelings of uncertainty, self-doubt [3], and work-related stress. Work-related stress results if an individual appraises themselves as ill-equipped to complete the task or cope with the job requirements [53]. Therefore, how an individual perceives their situation becomes important, and the stress response can be triggered by a threat (real or perceived) [56]. Consequently, stressors are determined by the individual’s appraisal of the situation and the perception they have regarding their ability to cope. The process of appraising a work stressor is subjective and includes both an emotional and cognitive component [57]. The ability to re-appraise situations is classified as a cognitive-based technique, and the underlying mechanisms were included in the intervention in the present study. As such, the purpose of the present study was to determine how participating in a 6-week wellbeing program changed attentional awareness, perceived stress, subjective wellbeing, job-affective wellbeing, burnout, and salivary cortisol levels for ECTs, if at all. Based on the literature reviewed and the pilot study, it was predicted that providing ECTs with a wellbeing program would result in a change in perceived stress, attention awareness, subjective wellbeing, and cortisol levels.

## 2. Materials and Methods

### 2.1. Participants

**Figure 1:**
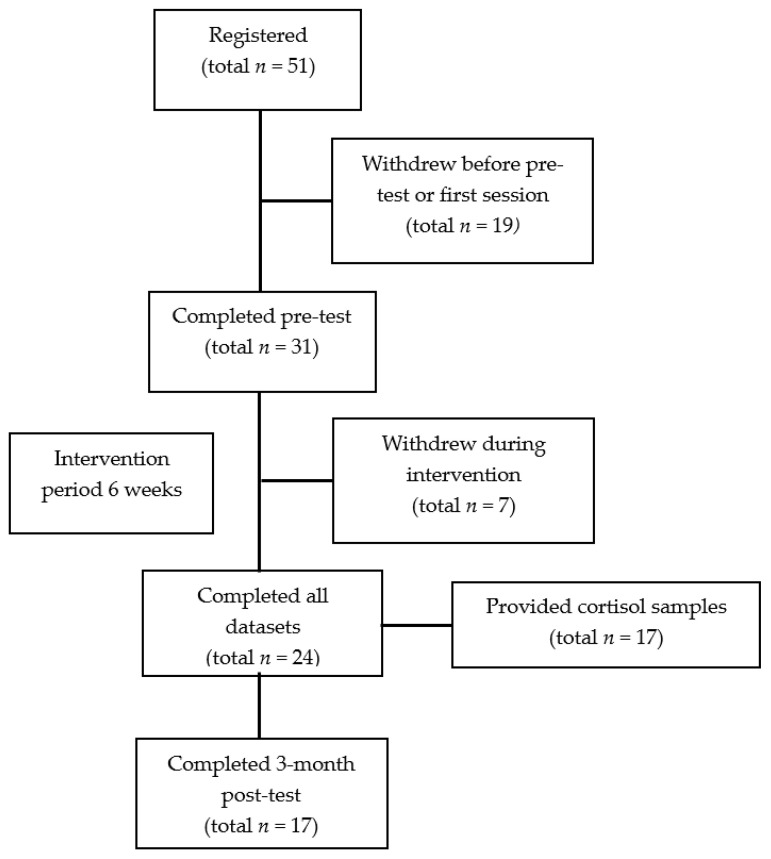
Participant flow diagram.

The self-selected participants registered for the study (*n* = 51); only the participants that completed the six-week intervention and pre- and post-measures were included in the final sample (*n* = 24). Participants that were within the early career period (1–5 years in-service) were included in the study. Due to funding restrictions, cortisol samples were included for 20 participants; however, three participants withdrew from the program; therefore, 17 participants completed salivary cortisol samples for the duration of the program. The post-intervention three-month follow up self-report measures were completed by 17 participants. The participant flow diagram (Figure 1) illustrates the number of participants at each stage in the intervention. Over 60% of the participants were entering the teaching profession with industry experience, which was reflected by 50% of the participants being under 37 years of age (range 23 to 58 years; M = 36.9, SD = 11.7). The average age of applicants for registration (teachers entering the profession) was 36.2 years in 2018, and 76.6% of registered teachers were female [58]. Therefore, the participant sample reflected the teaching population in Queensland, Australia, at the time of the intervention.

Forty-five percent of the participants were in their first year of teaching, and the participant characteristics are detailed in Table 2. The reasons for not attending or withdrawing from the program provided by the participants referred to the timing of the sessions, for example, conflicts with school-based events (e.g., staff meetings, coaching commitments), workload concerns, family responsibilities, and travel time required for attending sessions.

### 2.2. Measures

Mindful Attention Awareness Scale (MAAS). The MAAS [59] is designed to assess the receptive state of mind and awareness of the present, that is, the natural state of mindfulness. The 15-item self-report measure has been included in previous studies investigating integrating mindfulness training into teacher education programs (see [60,61,62]). The MAAS was selected due to the inclusion of indirect items, for example, *I rush through activities without being really attentive to them*. Indirect items relate to the characteristic of mindfulness as a natural state. State mindfulness is seen as a predictor of positive emotional states and self-regulated behaviour [59] (p. 822). The internal consistency levels range from 0.80 to 0.90 with high test–retest reliability, known-group validity, and convergent validity [59]. The MAAS is scored to produce an overall score rather than individual sub-scales or facets of mindfulness.

Personal Wellbeing Index (PWI). The PWI was developed from the Comprehensive Quality of Life Scale (ComQOL) ([63] by the International Wellbeing Group). The seven domains included in the ComQOL are reported as accurate measurements for subjective wellbeing and life satisfaction [64]. The seven domains are represented by eight items in the PWI: *safety*, *community*-*connectedness*, *health*, *relationships*, *future security*, *standard of living,* and *achievement in life.* The domain of *spirituality/religion* is an optional domain that was included in the present study. Each item is scored on an 11-point End-Defined Response Scale (i.e., 0 = no satisfaction at all to 10 = completely satisfied). The items can be scored to provide an overall subjective wellbeing score or individual scores for each domain [65]. In the present study, an overall subjective wellbeing score was calculated. To date, no known teacher wellbeing studies have included the PWI. The PWI measures the same construct for subjective wellbeing in both adult and child populations [66]. In Australia and overseas, the Cronbach alpha lies between 0.70 and 0.85.

Perceived Stress Scale (PSS). The PSS [67] is an established self-report measure used to assess psychological distress (e.g., *In the last month, how often have you felt that you were unable to control the important things in your life?*). The ten items were scored on a 5-point Likert scale (0 = never, to 4 = fairly often) and the higher the overall (summed) score, the higher the perceived stress level. The PSS coefficient alphas ranged from 0.77 to 0.78 [14]. The norm groups scores for females are 13.7 (*n =* 1406) and 12.1 (*n =* 926) for males from a US population sample (*n* = 2387) [68]. A score of 13 is reported as ‘average stress’, whereas scores of >20 are considered ‘high stress’. In previous research examining the use of MBIs for teachers, the PSS has been included as a measure for psychological stress [69,70], pre-service teacher wellbeing [60,61,71], and pre-service teacher stress [72,73].

Maslach Burnout Inventory Educators Survey (MBI-ES). MBI-ES is an established self-report measure included in research surrounding teacher stress [74] and MBI for teacher stress management [14,18]. The commercially available 22-item self-report measure assesses how often an individual experiences a feeling in relation to his/her teaching role, for example, *I feel emotionally drained from my work*. The items are scored on a 7-point Likert scale, and there are three sub-scales: depersonalisation (DP; *α* = 0.76), emotional exhaustion (EE; *α* = 0.87), and personal accomplishment (PA; *α* = 0.84) [75].

Job-related Affected Wellbeing Scale (JAWS). The JAWS is a self-report measure that includes a wide range of affective responses to explore how job stressors relate to various emotions and context-specific affective states experienced in relation to a job [76]. Teachers experience emotional reactions to work-related stress [77]. The participants are asked to indicate how often they have experienced an emotion in relation to their job in the past 30 days, for example, *My job made me feel at ease; my job made me feel angry*. The 30-items are scored on a 5-point Likert scale (1 = never to 5 = extremely often). The JAWS is predominantly included in research surrounding counterproductive work behaviours, emotional reactions to job conditions [78], and dysfunctional behaviour [79]. The total JAWS “has been found to be an excellent predictor of work-related stressors and strains and workload and coping” [80] (p. 364). The JAWS can be scored to produce a total scale score (*α* = 0.95), or a total for the negative emotion (*α* = 0.92) and positive emotion (*α* = 0.94) items, or four sub-scales (e.g., high pleasure–high arousal, high pleasure–low arousal, low pleasure–high arousal, low pleasure–low arousal) [76]. In the present study, the total negative emotion and positive emotion scores were calculated.

### 2.3. Intervention Design

The CI included in the present study is a multimodal secondary-level intervention that aimed to provide professional development units specifically designed to promote integrated wellbeing. The overarching approach of the program was to provide professional development to assist individuals’ identification of the stress response and the physiological and psychological impact of stress. This was highlighted as an important concept or strategy for pre-service and in-service teacher wellbeing [31,81,82,83]. The program was structured to increase awareness of the stress response and how stress can manifest in the body and behavioural changes, as well as providing specific strategies and techniques to reduce the stress response. The program content is provided in Table 3 and detailed in the section to follow.

#### 2.3.1. Psychological Wellbeing: Sessions One and Two

The theoretical content was linked to the teaching profession through reflection activities and guided meditation exercises. Ethical principles (*Yamas* and *Niyama*) were incorporated through individual reflection activities. The practical component of the unit included breathing techniques (*Pranayama*), postures (*Asana*), and guided meditation (*Pratyahara*) to specifically decrease the stress response. For example, ‘directing attention’ (FA meditation) was introduced as a technique to assist with decreasing the stress response, and the program content included the physical and psychological benefits of the technique.

#### 2.3.2. Physiological Wellbeing: Sessions Two and Three

The inclusion of diet and gut health was based on the link between mental health and nutrition [84] and the physiological impact of stress on the digestive system [85,86]. The reflection activities and guided meditation focused on the identification of behaviours and habits (professional and personal) that related to dietary choices (*Yamas*—interaction with others). The practical component highlighted the importance of breathing techniques (*Pranayama*) and postures (*Asana*) for improving digestion. The guided meditation (*Pratyahara*) extended the FA meditation techniques introduced in the previous unit and included Yoga Nidra meditation.

#### 2.3.3. Interpersonal Wellbeing: Sessions Five and Six

Social (interpersonal) wellbeing built on the foundation provided by the previous two units (increasing awareness and decreasing the impact of the stress response) to provide an overview of how the techniques promoted social wellbeing and the importance of social wellbeing for teachers (*Niyamas*—self-observation and *Yamas*—interaction with others). The guided meditation focused on compassion towards oneself and others, therefore incorporating ethical principles (*Niyamas* and *Yamas*). The reflection activities focused on professional and personal situations.

#### 2.3.4. Practical Component

The physical practice in each session was structured as a Hatha yoga class. The session began with guided breath awareness and relaxation breathing techniques in a restorative posture (e.g., constructive rest pose [87]) or a seated position. Yoga postures (*Asana*) can be divided into categories (e.g., twists, forward folds). The posture sequences focused on restorative, passive postures (e.g., a cat–cow sequence: gentle flexion and extension of the spine) and seated and supine poses. Passive static stretching was included through postures such as ‘supported bridge pose’ (*Setu Bandha Sarvangasana*) and active static stretching in forward fold posture (*Paschimottanasana*) and head-to-knee pose (*Janu Sirsasana*). Yoga postures assist with stretching connective tissue (fascia), and fibroblasts expand, which results in changes to extracellular fluid dynamics (e.g., swelling and inflammation), which can be created as a result of the stress response. Prolonged stretching is proposed to assist with the regulation of tissue fluid, immune function, and homeostasis [88]. The Yoga poses were selected to target stress-related conditions, for example ‘legs up the wall’ (*Viparita Karani*) and cross-legged twist (*Parsvasukhasana*) [53]. The Sun Salutation sequence (*Surya Namaskar*) was included in the longer physical practice sessions (weeks 4 to 6). The shorter sessions (weeks 1 to 3) included only supine and seated postures.

Each session concluded with a guided meditation, for example, Yoga Nidra or visualisation in corpse pose (*Sarvasana*) [89]. Yoga Nidra (‘yogic sleep’) is a form of guided relaxation typically included at the end of the physical practice, and it was included in the present study intervention. Yoga Nidra practice can range in length and can be completed as a stand-alone practice and include progressive muscle relaxation and/or visual imagery [53]. Changes in brain waves through electroencephalography (EEG) have been observed during Yoga Nidra practice, in particular alpha and theta waves [90]. The meditator becomes the neutral observer and can ‘withdraw’ (*Pratyahara*). Research has confirmed neurological changes that result during Yoga Nidra (see [91]) such as changes in ‘desire for action’ (blood flow in prefrontal cerebellar and subcortical regions) and endogenous dopamine release. Endogenous dopamine is a neurotransmitter involved in the regulation of motivation, mood, and pleasure, which can be reduced in the case of depression [38].

As previously detailed, yoga postures have been included in MBIs as a method for cultivating mindfulness [92], whereas the postures in the present program were included to specifically reduce the stress response on the body and target muscle groups that create physical tension.

#### 2.3.5. Feasibility and Fidelity

To ensure consistency, the same researcher facilitated the scripted sessions. The facilitator was an accredited yoga and meditation teacher with over 700 h of accredited training (accreditation held with registering bodies: Yoga Alliance—Level 1; Yoga Australia—Level 2). The theoretical component included a PowerPoint presentation and participant manual, and the practical component included scripted meditation and physical poses. The practical component (physical practice and guided meditation) increased in length each session to account for varying fitness levels. To increase accessibility, props (e.g., blocks, blankets, and yoga mats) were provided and adjustments made for clothing restrictions. The sessions were completed on the same day of the week for six consecutive weeks, outside of school hours (e.g., 4–6 p.m.). The participants were required to travel to a central location for the sessions.

### 2.4. Procedure

Prior to commencement, the study was approved by the Human Research Ethics Committee (HRECs). The study was guided by the Code for Responsible Conduct of Research from the National Statement on Ethical Conduct in Human Research (2007) [93]. Ethical clearance applications were submitted to each institution included in the study.

Because the use of yoga-based practices for teachers is a new area for research, there are limited study designs available. Similarly, there are no known self-report measures for yoga-based research (e.g., changes in proprioception and interoception). Previous research investigating the use of yoga-based techniques has included mindfulness-based self-report measures (e.g., the Five Facets of Mindfulness Questionnaire) and biological measures (e.g., blood pressure and salivary cortisol levels). The measures included in the study design reflect the existing practices in the field.

To maximise the participant sample, the project details were circulated in various professional networks and early career teacher conferences, and 49 Brisbane schools (P-6, P-12, 7–12) were contacted. The program was completed in Semester Two, 2018. One week before the scheduled start of the intervention, an information session provided detailed information about data collection, intervention structure, and time commitments. Participants registered for the intervention, and the baseline survey (MAAS, PSS, PWI, MBI-ES, and JAWS) was completed one week before the start of the intervention.

The baseline cortisol sample kit was supplied to the participants at the information session with samples taken the following day. The Oral Fluid Collector (OFC) swab collected 0.5 mL of oral fluid, and the swab contained a colour-changing volume adequacy indicator. The average collection time was between 20–50 s [94]. Single-use samples were taken after each session (following day) and returned each week. The samples were taken on the same day of the week for the duration of the program. Moreover, additional samples were taken from weeks 4 to 6 at the start and end of the session to collect data surrounding the immediate impact of the full one-hour physical practice.

**Figure 2:**
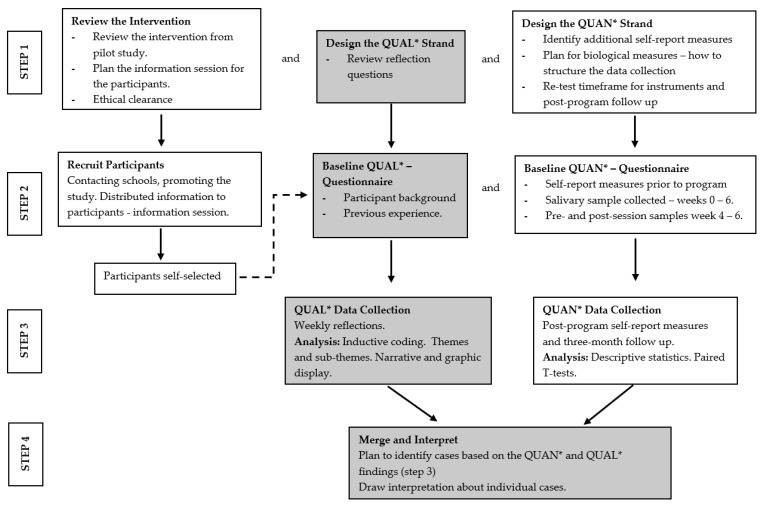
Study design flow chart * QUAL—qualitative; * QUAN—quantitative.

The study design flowchart (Figure 2) illustrates the convergent mixed-methods design. The reflections were administered via email and completed for the duration of the program. The weekly reflection was completed at the start of each session and the post-session reflection was completed at the end of each session. The qualitative datasets included descriptive (what was experienced) and subjective (how was it experienced) questions, providing an opportunity to evaluate the program. The post-program survey was sent via email one week after the final session. A follow-up survey was sent via email three months after the conclusion of the program. Due to the large volume of data collected, the qualitative datasets (i.e., shaded boxes in Figure 2) will not be reported in this paper.

### 2.5. Data Preparation and Analysis

#### Quantitative Data

Responses to the self-report measures were exported from Qualtrics to IBM Statistical Software SPSS Version 26 (IBM Corp., Armonk, NY, USA), cleaned, and re-coded as per the scale author’s guidelines. The risk of data entry error was reduced through response collection via Qualtrics. Descriptive and inferential statistics were completed to test for normality (e.g., histogram and Q-Q plot of difference), and the data met the required assumptions for completing a paired-samples t-test. Due to the size of the participant sample for salivary cortisol levels (*n* = 17), normality was tested using a Shapiro–Wilk test. The pre- and post-data were prepared using the same procedure, and the responses were matched via the participant codes. The Perceived Stress Scale, Mindful Attention Awareness Scale, and Personal Wellbeing Index were coded to produce a single score for perceived stress, mindful attention awareness, and subjective wellbeing. The MBI-ES produced three sub-scale scores for depersonalisation, emotional exhaustion, and personal accomplishment. The JAWS scale was scored to produce two subscales, positive emotions and negative emotions. A paired-sample *t*-test was completed to compare the pre- and post-program scores.

The components required for the cortisol sample analysis were the IPRO Cube Reader and IPRO (Lateral Flow Device) LFD cassette, specific to the analyte under investigation (cortisol nM). SOMA Bioscience (Wallingford, United Kingdom) provides an IPRO Cube Reader Manual that outlines the instructions for sample collection, analysis, and relevant details surrounding the use of the product. A timer was used to ensure consistency with the testing procedure. The ‘immediate measurement’ procedure was completed for analysing each sample [95]:

1. OFC buffer bottles (containing OFC swab) were agitated for 2 minutes.
2. Two drops of the buffer and saliva mixture were added to the cortisol LFD.
3. The IPRO Cube Reader was used to scan the LFD at exactly 10 minutes.

The IPRO Cube Reader provides cortisol values that range from <1 to >40 nM. The results were recorded in an Excel spreadsheet for each sample, and participant codes were used to identify the individual samples over the duration of the program. The readings for each week were summed for the total participants (*n* = 17) and the mean calculated for each week.

## 3. Results

### 3.1. Internal Consistency and Descriptive Statistics

As illustrated in Table 4, there was high internal consistency (Cronbach’s alpha above 0.70) across the self-report measures in the pre-program samples and in the post-program sample with the exclusion of the sub-scale ‘depersonalisation’ in the MBI-ES.

The norm population perceived stress scores for ages 30–44 is 13.0 [68]. The PSS author’s report a score of 13 is classified as ‘average stress’, whereas scores of >20 are classified as ‘high stress’. In the present study, the PSS score pre-program distribution showed approximately 50% of the participants reporting a score above 19.5. Approximately 25% (upper quartile range—Q3) of the participants reported a score above 27.5, and approximately 25% (lower quartile range—Q1) reported a score below 15.5. Therefore, 75% of the participant sample reported a score above the norm (‘average stress’). The post-program results were approximately symmetrical (i.e., normal distribution) and indicated 50% of the participants reported a score of 16.5 or above, the upper 25% (Q3) of the participants reported a score above 19.5, and the lower 25% (Q1) reported a score below 13. The three-month follow-up survey (*n* = 17) resulted in 50% of the participants reporting a score of 13 or above, the top 25% (Q3) reported a score of 20 or above, and the lower 25% (Q1) reported a score of 10 or below, indicating a further decrease in perceived stress.

The normative mean range for the MAAS is reported as 4.2 (SD = 0.69) in community adults (*n* = 436) and 3.83 (SD = 0.70) in college-aged students (*n* = 2277) [59]. Approximately 50% of the participants in the present study reported a score of 3.5 or higher at the baseline, 4.1 post-program, and 3.9 at the three-month follow up. The participants in the present study were below the reported mean for the PWI with a pre-program mean of 52 and 57.4 post-program. The normative mean range for the PWI for Western countries is 70–80 points, and for Australia 73.4–76.4 points [65].

The reported norm for negative emotions in the JAWS (*n* = 166) was M = 23 and SD = 7.7, and for positive emotions (*n* = 166) M = 30.2 and SD = 9.3 [78]. The participant sample in the present study reported below the norm mean for positive emotions and above the norm mean for negative emotions pre- and post-program. For the MBI-ES, the participants were above the reported norm for Primary and Secondary teachers (*n* = 4163) for emotional exhaustion (M = 21.25, SD = 11.01) and personal accomplishment (M = 33.54, SD = 6.89) and lower for depersonalisation (M = 11.00, SD = 6.19) [75].

### 3.2. Paired-Samples *t*-Tests: Pre- and Post-Intervention

The Shapiro–Wilk test showed the probability of normality for MAAS W (24) = 0.94, *p* = 0.24, PSS W(24) = 0.96, *p* = 0.63, PWI W(24) = 0.98, and *p* = 0.92; therefore, normality was assumed (*p* > 0.05). Due to the sample size (*n* > 20) and assumed normality, based on the Shapiro–Wilk test, paired-samples *t*-tests (Table 5) were completed to establish the change in attention awareness, perceived stress, subjective wellbeing, job-related affective wellbeing, and burnout.

The paired-sample *t*-test mean scores for the PSS ($\bar{x}$*_d_* = 4.5, *s_d_* = 5.4) pre-program (M = 21.13, SD = 7.07) and post-program (M = 16.63, SD = 4.5) was statistically significant, *t*(23)= 4.019, *p* = 0.001 (*p*-value less than 0.05). The large effect size (*d* = 0.82) [96] indicates that the results support the contention that perceived stress levels decreased pre- to post-program. The 95% confidence interval for the mean difference was (2.1, 6.8), indicating a moderate confidence interval. The confidence interval suggests that the post-program mean score could be 6.8 points higher or 2.1 points lower than the pre-program score. The same analysis was completed for the three-month follow up to provide insight into ongoing changes or impact; however, the decreased sample size does present a limitation. The three-month follow up (M = 15.41; SD = 7) and the post-program scores (conditions *t* (16) = 1.1, *p* = 0.28) support that there was not a significant change between the post-program score and the three-month follow up, suggesting that the changes had been maintained. This was supported by the difference in mean scores ($\bar{x}$*_d_* = 1.17, *s_d_* = 4.3), which did not produce a large effect size (*d* = 0.26) [96].

The mean scores for mindful attention awareness pre-program (M = 3.55, SD = 0.79) and post-program (M = 4.0, SD = 0.67) with the conditions *t*(23) = −3.54, *p* = 0.002) also suggest an increase in mindful attention awareness scores pre- and post-program. However, there was a narrow range for the 95% confidence interval for mindful attention awareness scores [−0.70, −0.18]. The difference in mean scores ($\bar{x}$*_d_* = −0.44, *s_d_* = 0.61) indicated a large negative effect size (*d* = −0.72), suggesting an increase in MAAS scores post-program. The three-month follow up (M = 4.01; SD = 0.67) and the post-program scores (conditions *t*(16) = −0.75, *p* = 0.46) support that there was not a significant change between the post-program score and the three-month follow up, suggesting that the changes had been maintained. Similarly, the difference in the mean scores ($\bar{x}$*_d_* = 0.058, *s_d_* = 0.32) did not produce a significant effect size (*d* = −0.1).

The mean scores for the PWI scores pre-program (M = 52, SD = 9.3) and post-program (M = 57.4; SD = 11.53) indicated a statistically significant change, *t*(23) = −3.05, *p* = 0.006). The difference in mean scores ($\bar{x}$*_d_* = −5.4, *s_d_* = 8.6) produced a medium effect size (*d* = −0.62). The three-month follow up (M = 57.3; SD = 11) and the post-program scores (conditions *t*(16) = 0.13, *p* = 0.89) support that there was not a significant change between the post-program score and the three-month follow up. The difference between the mean scores ($\bar{x}$*_d_* = 0.29, *s_d_* = 8.9) produced a small effect size (*d* = 0.03) [96].

The JAWS scores pre- and post-program did not produce statistically significant changes for either sub-scales (positive emotions and negative emotions) (see Table 5). However, there was an increase in positive emotions and a decrease in negative emotions.

The MBI-ES did not produce a significant change in the three sub-scales, with a summary of the data presented in Table 5.

Therefore, the results suggest the six-week CI resulted in a decrease in perceived stress and an increase in attention awareness and subjective wellbeing. The positive emotions from the Job-Affective Wellbeing Scale increased post-program, and the negative emotions decreased; however, there was not a statistically significant change. Similarly, there was a decrease in the sub-scales of emotional exhaustion and depersonalisation from the Maslach Burnout Inventory Educator Survey; however, the personal accomplishment sub-scale decreased pre- to post-program. The changes presented in the results warrant further investigation, including a larger participant sample size.

### 3.3. Weekly Salivary Cortisol Levels: Waking (CAR) and Resting Levels

**Figure 3:**
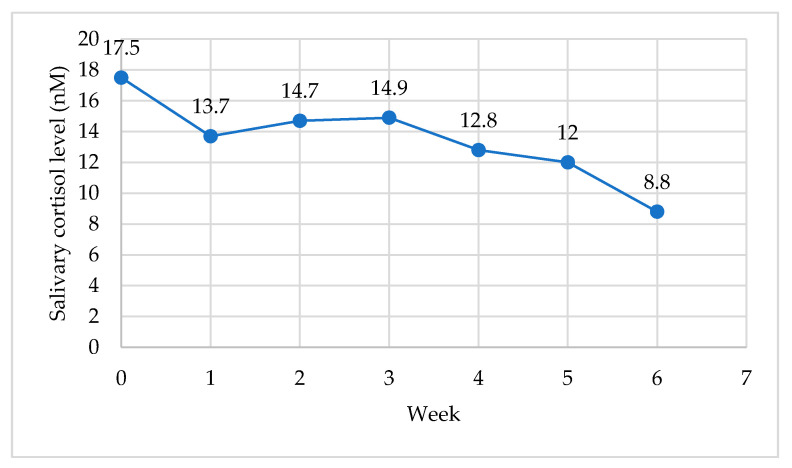
Waking (CAR) salivary cortisol levels from baseline to week 6 of the program (*n* = 17).

**Figure 4:**
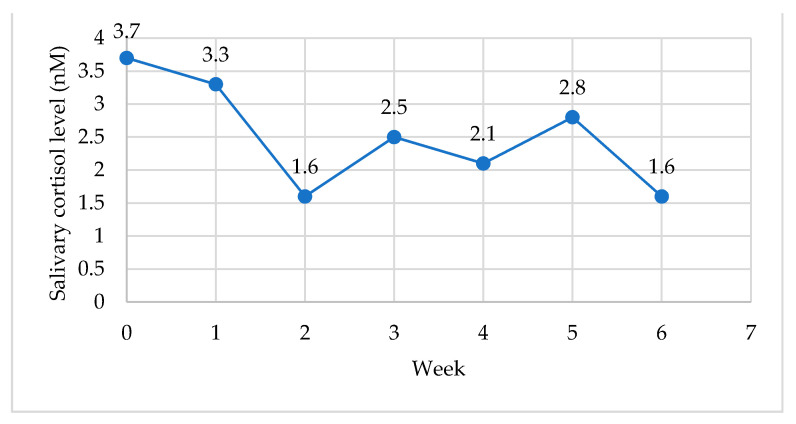
Resting salivary cortisol levels from baseline to week six of the program (*n* = 17).

The biological measures reported include the weekly salivary cortisol samples for cortisol awakening rise (CAR), resting level and pre- and post-session levels. The weekly cortisol samples were collected from the baseline (week 0) to week 6. To illustrate the change over the duration of the intervention, the weekly mean values for the waking (morning) salivary cortisol levels are illustrated in Figure 3, and the resting (evening) levels are illustrated in Figure 4.

#### Paired-Sample t-Tests Pre- and Post-Program

A Shapiro–Wilk test showed the probability of normality for the CAR (waking) dataset, W (17)= 0.93, *p* = 0.30, and resting dataset, W(17) = 0.97, *p* = 0.91; therefore, normality was assumed (*p* > 0.05). Paired sample *t*-tests were completed to compare the measure scores pre- (week 0) and post-program (week 6) and pre- and post-session for weeks 4 to 6. A summary of the paired sample *t*-test analysis is provided in Table 6.

The mean scores for cortisol awaking rise (CAR) pre-program (week 0) (M = 17.3, SD = 4.2) and post-program (week 6) (M = 9.1, SD = 4.2) suggest a significant decrease in CAR, *t* (14) = 6.3, *p* = 0.001). The difference in mean scores ($\bar{x}$*_d_* = 8.14, *s_d_* = 4.97) indicated a large effect size (*d* = 1.66), that is, the CAR levels at week 6 were over 1.5 standard deviations lower than the CAR levels at baseline. The mean scores for resting salivary cortisol levels pre-program (week 0) (M = 3.5, SD = 1.5) and post-program (week 6) (M = 1.9, SD = 1.1) suggest a significant decrease in resting salivary cortisol levels pre- and post-program, *t*(14)= 3.3, *p* = 0.005. The difference in mean scores ($\bar{x}$*_d_* = 1.5, *s_d_* = 1.76) and large effect size (*d* = 0.85) indicate a change in resting cortisol levels from baseline to week 6.

### 3.4. Pre- and Post-Session Salivary Cortisol Levels

**Figure 5:**
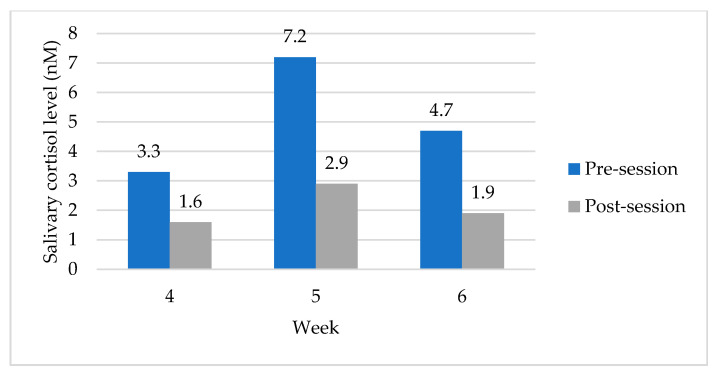
Pre- and post-session salivary cortisol samples (*n* = 17).

Salivary cortisol samples were collected at the start (4 p.m.) and end (6 p.m.) of the sessions for weeks 4 to 6 (Figure 5). The post-session sample for each week was below the reported mean for each week. For example, the post-session sample for week 4 was M = 1.56, and the resting level for week 4 (see Table 5) was M = 2.1. Similarly, the resting level for weeks 5 and 6 was above the post-session level.

#### Paired-Sample *t*-Tests Pre- and Post-Program

The mean scores for salivary cortisol levels pre-session for week 4 (M = 3.44, SD = 1.36) and post-session (M = 1.56, SD = 0.65) conditions, *t* (14) = 6.3, *p* = 0.001) suggest a significant decrease in salivary cortisol levels pre- and post-session. The difference in mean scores ($\bar{x}$*_d_* = 1.88, *s_d_* = 1.14) indicated a large effect size (*d* = 1.64) [96] and suggested that the post-session cortisol level was over 1.5 standard deviations lower than the pre-session cortisol level. Similarly, week 5 pre-session (M = 7.1, SD = 4.8) and post-session (M = 2.7, SD = 2.45), *t*(14)= 4.3, *p* = 0.001, and week 6 pre-session (M = 4.4; SD = 2.6) and post-session (M = 1.8; SD: 0.66), *t* (16) = 4.2, *p* = 0.001, suggesting a significant decrease in salivary cortisol levels pre- and post-session. The difference in mean scores for week 5 ($\bar{x}$*_d_* = 4.46, *s_d_* = 3.94) and week 6 ($\bar{x}$*_d_* = 2.6, *s_d_* = 2.54) produced a large effect size (week 5 *d* = 1.13; week 6 *d* = 1.02) indicating the post-session cortisol levels were over one standard deviation lower than the pre-session levels.

## 4. Discussion

As hypothesised in the introduction, the participants in the intervention (*n* = 24) experienced a decrease in perceived stress (PSS) and an increase in attention awareness (MAAS) and subjective wellbeing (PWI). There was not a significant change in burnout (MBI-ES) or job-related wellbeing (JAWS).

The participants in the program reported a ‘high stress’ score (above 20) [67] pre-program, which decreased by 21% post-program. The results are consistent with the findings from the pilot study [97] and MBIs for teachers (e.g., [14,16]). The three-month post-program follow up survey suggested a further decrease in perceived stress, suggesting the changes had been maintained and the participants experienced a further decrease in perceived stress.

The baseline survey was completed in the second week of a 10-week school term in the second semester of the academic year. Therefore, the PSS referred to the previous month during which the participants would have been on holidays and starting the school term. During the holiday period and at the commencement of the term, ECTs may experience a phase of *anticipation* that is linked to feelings of anxiety and excitement, which can shift to *survival* once workload increases [98]. The post-program PSS was completed in week 8 of the ten-week term during the assessment and reporting period. This time of the term could be considered a high-stress period for teachers. The reporting and marking period has previously been referred to as a *disillusionment* phase when morale can drop, and the end of the academic year is a period of *reflection* when ECTs reflect on their abilities and career choice [98]. The decrease in PSS scores in the present study indicated that it may be beneficial to enable ECTs with strategies to assist with positive reappraisal after challenging situations. Decreased reactive behaviour has been associated with decreased perceived stress [26]. It is proposed that student–teacher interactions may be improved due to decreased reactive responses from teachers, that is, teachers appear less irritable or easily triggered by student behaviour [12].

Increasing attention awareness (volition) through processes such as meditation practices and decreasing the stress response through physiological mechanisms (e.g., yoga postures and breathing practices) has been explored in previous research surrounding the use of MBIs in clinical settings for the treatment of stress-related disorders and the integration of MBIs and CIs in school contexts. The findings in the present study indicated a significant (*p* = 0.002) increase in MAAS scores pre- and post-program, which were maintained at the three-month post-program survey. The lower mean MAAS score pre-program could relate to the above-average perceived stress levels. It has been previously suggested that there is a negative correlation between MAAS and PSS [97]. The increase in MAAS was evidenced in the findings from the pilot study, and changes in mindfulness (measured using the MAAS) have been reported in studies investigating the use of MBIs for teachers (e.g., [13]). Likewise, increased mindfulness measured using the Five Facets of Mindfulness Questionnaire (FFMQ; [99]) has been reported in MBIs and modified Mindfulness-based Stress Reduction (mMBSR) programs for educators (e.g., [14,16,18,19,26]). In the field of research surrounding MBIs and CIs for educators, mindfulness is proposed as a strategy that assists teachers with cultivating present-centred awareness (state and trait mindfulness, including body awareness) [11].

Previous research supports the use of mindfulness-based strategies to reduce burnout (measured with the MBI-ES). For example, findings from MBIs for educators have indicated a decrease in educator burnout (e.g., [14,26,29]), whereas other research suggests a significant change on the sub-scales (e.g., emotional exhaustion; [18] and depersonalisation; [30]). In the present study, the MBI-ES was scored for the three sub-scales, not as a collective ‘score’ of burnout. The participants reported a decrease in emotional exhaustion and depersonalisation; however, the results did not produce a significant change. The MBI-ES has previously been included in mMBSR programs and MBIs (e.g., [100]) that did not produce a significant result (e.g., [19]). It may be that mindfulness-based and yoga-based approaches are not as influential on educator burnout as contextual factors such as job demands and availability of resources and support. An additional consideration is the career period; ECTs at the start of their career may not be experiencing similar feelings of burnout as mid-career or veteran teachers. Similarly, personal and family commitments may be an influential factor surrounding burnout.

The JAWS has not been included in previous research for MBIs or CIs for educators. The results did not produce a significant change in the positive or negative emotions sub-scale; however, the trend indicated an increase in positive emotions and decrease in negative emotions. Previous research has evaluated the use of MBIs for educators with reference to regulation of emotions at work indicating increased efficacy of emotion regulation (e.g., [29]).

There are limited previous studies investigating MBIs and CIs for educators that include measures for subjective wellbeing. MBIs have been adapted from clinical trials and interventions specifically designed for the treatment of stress-related conditions rather than the promotion of wellbeing. For example, self-report measures included in previous trials for MBIs assessed changes in mindfulness in conjunction with anxiety, depression, negative and positive effect, and rumination [13]; self-compassion, physical symptoms, sleep quality, and self-efficacy [19]; time urgency [26]; and forgiveness and occupational stress [21]. Changes in subjective wellbeing (measured on the Warwick–Edinburgh Mental Well-being Scale; [101] were reported in an 8-week MBI for secondary teachers [16] and a 6-week MBI for pre-service teachers [61]; subjective wellbeing was measured on the WHO-Five [102]. The findings from the present study similarly indicated an increase (*p* = 0.006) in subjective wellbeing pre- and post-program, and the changes were maintained at the three-month follow-up. Perceived stress may be more influential on subjective wellbeing than attention awareness (mindfulness) [97]. Therefore, it could be suggested that the decrease in PSS scores reflects the increase in PWI scores (pre- and post-program), which were maintained at the three-month follow-up period. However, wellbeing is a complex, multifaceted concept that is influenced by an assortment of factors, not limited to emotions, brain activity, neurochemistry, and environmental factors such as social support and health resources [103].

The main drivers in the stress response are the SNS and epinephrine; the role of cortisol is to regulate the response [56]. When the acute stress response becomes chronic, there is potential for the development of disease [104]. The acute (and chronic) release of neuroendocrine mediators affects the immunocompetent cells in the immune system. Short-term changes to immune function are part of an adaptive process; however, disruption to the neuroendocrine axis can increase the risk of chronic inflammatory disease and susceptibility to viral infections [105]. CAR is the increase in cortisol before waking and is a part of the circadian rhythm for cortisol in the body. There is a large increase (50–100%) within the first hour after awakening, followed by a steady decrease over the day, resulting in the lowest level before sleep. CAR is necessary to transition from the sleep state to an awake state. CAR has been associated with psychosocial variables, health, and stress and is often viewed as an adaptive response that occurs due to the anticipation of the upcoming day [106]. Higher CAR levels have been linked to chronic fatigue syndrome [107], whereas lower CAR levels have been linked to post-traumatic stress disorder [108].

As previously outlined, it is difficult to isolate a specific mechanism that results in a reduction of the physiological or psychological stress response, because the techniques are practised in unison; however, research has indicated that there are differences between the benefits experienced based on the techniques practised. For example, integrated yoga (including ethical principles) may produce a reduction in cortisol in comparison to yoga as exercise (postures and breathing practices only) [39].

Threat detection and perceived stress influence the HPA axis and the release of cortisol, and the PSS results in the present study support the physiological findings. There was a significant decrease (*p* = 0.001) in cortisol awakening rise (CAR) pre-program (week 0) and post-program (week 6), and the trend over the duration of the intervention indicated a decrease in CAR. Similarly, the mean scores and large effect size for resting salivary cortisol levels pre- and post-program suggest a significant decrease (*p* = 0.005). The changes in perceived stress may relate to the decrease in CAR in the present study. Previous research suggests perceived stress is linked to HPA-axis feedback; for example, teachers with higher levels of perceived stress had higher salivary cortisol levels one hour after waking, and teachers with a lower CAR level experienced higher burnout levels [109]. Physiological indicators of stress (e.g., cortisol) included in previous MBIs for educators (e.g., [29]) have not produced a significant effect. However, Harris and colleagues [30] suggested that a 16-week yoga-based CI for teachers created a preventive effect on CAR, reporting changing (increased) CAR for the control group and stable CAR in the intervention group. A decrease in morning cortisol levels was reported for an 8-week modified-Mindfulness-based Stress Reduction Program (mMBSR) [18]; however, the collection period was for three consecutive days during the intervention, whereas the design of the present study included weekly cortisol (CAR and resting) samples the day after the program session for the duration of the intervention. The aim was to establish if the session created a change in the CAR the morning after the evening session. Previous research has indicated that a 7-week integrated yoga intervention (in comparison to yoga as exercise) can produce a significant change in CAR over the duration of the intervention [39]; however, it is not known if the sample collection occurred the morning after the session.

The practical component in the intervention was included to assist with reducing the three layers of tension that accumulate, muscular (physical), emotional, and mental tension [35] (Table 1). Diaphragmatic breathing and voluntary control of breathing patterns [48] and practicing physical postures with non-reactive awareness [45] can result in vagal stimulation, which promotes GABA levels in the brain and consequently downregulates allostatic load, the sympathetic nervous system (SNS) stress response, and HPA activation [48]. In the present study, pre- and post-session samples were collected for the sessions that included the longer physical component of 45–60 min of physical practice (weeks 4 to 6). The aim was to identify if the sessions provided an immediate effect on salivary cortisol levels. The results suggested a significant effect for the pre- and post-session salivary cortisol levels. Salivary cortisol testing for pre- and post-session effect has not been included to date in MBIs or CIs for educators. Previous mind–body research including pre- and post-session cortisol salivary levels indicated that a 90-min yoga class created a significant decrease in salivary cortisol levels [41]. Similarly, research investigating HPA activity has suggested that after 120 min of yoga practice, cortisol levels decreased [36]. Top-down and bottom-up mechanisms can regulate the stress response. For example, a body scan meditation practice includes both mechanisms, that is, peripheral sensory vessels stimulated by visceral activities (e.g., decreased muscle tension) (bottom-up mechanism) and focused attention and conscious relaxation (top-down mechanism) [110]. Yoga has been viewed as a form of ‘self-massage’ due to the movement of the body into and out of postures, which stimulates the pressure receptors in the skin, vagal activity, HPA axis, and reduction in cortisol. It is proposed that the underlying mechanisms for the effects of yoga are similar to massage therapy, and the physiological and biochemical changes are expected to accompany a decrease in feelings of depression, immune problems, and somatic pain [111,112].

The project was completed as part of a Higher Degree by Research (Doctor of Philosophy), and convenience (non-probability) sampling [113] relied on the participants volunteering for the project. The challenges of convergent mixed-methods design were considered, and the evaluative criteria proposed by Teddlie and Tashakkori [114] were reviewed throughout the study. The datasets (self-report measures) collected were from one participant sample in the same phase, and the absence of a control group was addressed through the inclusion of multiple datasets, that is, the inference quality was strengthened through the inclusion of multiple datasets [114]. The different sample sizes (biological measures and self-report measures) presented a limitation. The participant sample provided a homogenous sub-group of the broader teaching population (i.e., ECT with one to five years of experience). The small sample size does present a limitation; however, previous studies, including pilot MBIs for educators, similarly include small participant samples (e.g., [18,19,28]). The results from the present study warrant further investigation with a larger participant sample.

## 5. Conclusions

An overarching concept in the present study relates to the impact of the stress response and integrated wellbeing, in particular, the relationship between the stress response (physiological and psychological), stress management techniques (mechanisms), and subjective wellbeing. The authors of [103] outlined that increasing flourishing in a population may serve as a preventative measure and a proactive approach as opposed to focusing on treating disease. This is relevant to the present study because previous interventions for educators have focused primarily on mindfulness-based approaches, which were traditionally developed for clinical applications and the treatment of stress-related conditions. However, yoga is more often associated with fitness and health as opposed to disease and treatment [92]. Limited studies examine the use of yoga-based techniques for stress management or wellbeing interventions for educators. The present study aims to make a valuable contribution to this area, and the quantitative findings reflected the existing research surrounding the use of MBIs and CIs for educators. However, the data collection methods for the biological measures provided additional findings not previously included in MBIs and CIs for educators.

## Figures and Tables

**Table 1:** Techniques from the System of Yoga * and stress management.

Technique	Mechanism	Layers of Tension	Output	
			Decrease	Increase
Meditation Practices (*Samyama*)	Attentional stability Response inhibition Meta-awareness	Mental	Habitual maladaptive tendencies.	Executive monitoring systems.
Ethical Principles (*Yama*, *Niyama*)	Introspection Non-reactive awareness	Emotional	Rumination Emotional reactions.	Prosocial behaviour.
Postures (*Asana*) Breathing Practice (*Pranayama*)	Interoceptive and proprioceptive awareness. Vagal tone.	Physical	Cortisol Inflammation Tension HPA-axis activation	PSN activation.

* The limbs *Pratyahara* (sensory withdrawal), *Dharana* (concentration), *Dhyana* (meditation), and *Samadhi* (self-realisation) are represented under Meditation Practices—*Samyama*.

**Table 2:** Participant characteristics (*n* = 24).

Characteristic	All Participants %	*n*
Female (%)	91.7	22
Male (%)	8.3	2
Fulltime (%)	75	18
Part-time (%)	25	6
First career (%)	37.5	9
Industry experience prior to teaching (%)	62.5	15
Dependents: Yes (%)	10	41.7
Dependents: No (%)	14	58.3
First year teaching (%)	45.8	11
Second year teaching (%)	12.5	3
Three to five years’ teaching (%)	41.6	10
Lower Primary: Prep–Grade 2 (%)	26.1	6
Upper Primary: Grade 3–6 (%)	56.5	13
Secondary: Grade 7–9 (%)	17.4	4
Secondary: Grade 10–12 (%)	0	0
Secondary: Grade 7–12 (%)	4.2	1

**Table 3:** Theoretical and practical program content.

Topic	Week	Theoretical Content	Physical Practice (Minutes)	Activities
Psychological Wellbeing	1	The impact of stress	20–25	Instant relaxation techniques: breathing practices, visualisation Restorative poses and sequences. Supported inversions. Guided meditation: Yoga Nidra, Compassion meditation, positive experiences. Gentle stretching (postures). Reflection activities Group discussion
	2	The benefits of relaxation	30	
Physiological Wellbeing	3	The importance of exercise	30–45	
	4	Diet and stress	60	
Social Wellbeing	5	Self-compassion	60	
	6	Professional relationships	60	
	Home practice: 3–20-min guided meditation (pre-recorded audio track) 10–20-min yoga sequence (pre-recorded track) Breathing practices (practice length determined by the individual)			

**Table 4:** Cronbach’s alpha calculations for the self-report measures (*n* = 24).

Scale	Cronbach’s Alpha A	
	Pre-Program	Post-Program
Mindful Attention Awareness (MAAS)	0.89	0.89
Perceived Stress (PSS)	0.92	0.80
Personal Wellbeing Index (PWI)	0.86	0.89
Maslach Burnout Inventory (MBI-ES) Emotional Exhaustion Depersonalisation Personal Accomplishment	0.91	0.75
	0.90	0.59
	0.73	0.90
Job-related Affective Wellbeing (JAWS) Positive emotions	0.86	0.90
Negative emotions	0.89	0.88

**Table 5:** Paired sample *t*-test results summary *.

	Pre-Program		Post-Program		Paired *t*-Test				
Scale	Mean	SD	Mean	SD	${\bar{x}}_{d}$	*s_d_*	$\hat{d}$	*t*	*p*-Value
MAAS	3.55	0.79	4.0	0.67	−0.44	0.61	−0.72	−3.54	0.002
PSS	21.13	7.07	16.63	4.5	4.5	5.48	0.82	4.01	0.001
PWI	52	9.3	57.4	11.53	−5.4	8.6	−0.62	−3.05	0.006
MBI-ES Emotional Exhaustion	27.63	11.08	24.92	10.14	2.7	9.38	0.28	1.4	0.17
MBI-ES Depersonalisation	7.13	8.20	6.50	5.46	0.62	5.32	0.11	0.57	0.57
MBI-ES Personal Accomplishment	36.21	5.16	35.29	7.03	0.91	6.7	0.13	0.66	0.51
JAWS—Positive	28.33	5.69	29.63	6.02	−1.29	4.5	−0.26	−1.38	0.18
JAWS—Negative	24.58	6.32	23.17	6.12	1.4	5.08	0.27	1.36	0.18

* including mean, standard deviation pre- and post-program; mean of the difference ($\bar{x}$*_d_*), standard deviation of the sample of differences (*s_d_*), effect size ($\hat{d}$), t-value, and *p*-value (df = 1(23)).

**Table 6:** Paired sample *t*-test results summary for Time One (pre-program or pre-session) and Time Two (post-program or post-session) *.

	Time One		Time Two		Paired *t*-Test				
	Mean	SD	Mean	SD	${\bar{x}}_{d}$	*s_d_*	$\hat{d}$	*t*	*p*-Value
Waking cortisol level	17.26	4.22	9.12	4.27	8.14	4.97	1.66	6.34	0.001
Resting cortisol level	3.48	1.56	1.98	1.16	1.5	1.76	0.85	3.29	0.005
Session Four	3.44	1.36	1.56	0.65	1.88	1.14	1.64	6.37	0.001
Session Five	7.18	4.81	2.72	2.45	4.46	3.94	1.13	4.38	0.001
Session Six	4.44	2.62	1.84	0.66	2.6	2.54	1.02	4.2	0.001

* mean of the difference ($\bar{x}$*_d_*), standard deviation of the sample of differences (*s_d_*), *t*-value, and *p*-value (*df* = 1(16)).

## Data Availability

Data available on request due to restrictions. The data are not publicly available due to the conditions specified in the ethics application.
